# Dispersal-competition tradeoff in microbiomes in the quest for land colonization

**DOI:** 10.1038/s41598-018-27783-6

**Published:** 2018-06-21

**Authors:** Francisco Dini-Andreote, Jan Dirk van Elsas, Han Olff, Joana Falcão Salles

**Affiliations:** 1 Microbial Ecology cluster, Genomics Research in Ecology and Evolution in Nature (GREEN), Groningen Institute for Evolutionary Life Sciences (GELIFES), University of Groningen, Groningen, The Netherlands; 2 Conservation Ecology group, Genomics Research in Ecology and Evolution in Nature (GREEN), Groningen Institute for Evolutionary Life Sciences (GELIFES), University of Groningen, Groningen, The Netherlands

## Abstract

Ancestor microbes started colonizing inland habitats approximately 2.7 to 3.5 billion years ago. With some exceptions, the key physiological adaptations of microbiomes associated with marine-to-land transitions have remained elusive. This is essentially caused by the lack of suitable systems that depict changes in microbiomes across sufficiently large time scales. Here, we investigate the adaptive routes taken by microbiomes along a contemporary gradient of land formation. Using functional trait-based metagenomics, we show that a switch from a microbial ‘dispersal’ to a ‘competition’ response *modus* best characterizes the microbial trait changes during this eco-evolutionary trajectory. The ‘dispersal’ *modus* prevails in microbiomes at the boundary sites between land and sea. It encompasses traits conferring cell chemosensory and motile behaviors, thus allowing the local microbes to exploit short-lived nutritional patches in high-diffusion microhabitats. A systematic transition towards the ‘competition’ *modus* occurs progressively as the soil matures, which is likely due to forces of viscosity or strain that favor traits for competition and chemical defense. Concomitantly, progressive increases in the abundances of genes encoding antibiotic resistance and complex organic substrate degradation were found. Our findings constitute a novel perspective on the ecology and evolution of microbiome traits, tracking back one of the most seminal transitions in the evolutionary history of life.

## Introduction

The marine-to-terrestrial transition has driven the adaptation of the major forms of life on Earth. In particular, the microbiomes of coastal habitats have been subjected to such transitions as from early evolutionary time^1^, when ecophysiological trait selection allowed microorganisms to thrive under local conditions characterized by progressive increases in the levels of heterogeneity – i.e. changes in microhabitat structure with concurrent increases in oxygenation levels. In such local microhabitats, interactive processes between microbes (e.g. competitive and mutualistic interactions) were presumably on the basis of the emergence of communities with different prevailing traits^2^. However, the eco-evolutionary mechanisms underlying microbiome adaptation to terrestrial life are, thus far, greatly understudied, mainly because of (*i*) the lack of a fossil record for most microbial groups, and (*ii*) the absence of studies probing this eco-evolutionary transition along analogous contemporary gradients of soil formation.

From a contemporary ecological perspective, gradients of soil formation (i.e. soil chronosequences), such as those found in coastal ecosystems, are well-suited systems to examine the drivers of trait-based adaptation of local microbiomes across comprehensive time scales. This approach particularly invokes the theme of ecological succession, where communities change over time in a dynamic, and, most importantly, systematic and progressive manner^3,4^. Gradients of naturally forming soil chronosequences can expand over hundreds to thousands of years^5^. In these systems, the temporal scale is based on the assumption of space-for-time substitutions. That is, ecological succession is investigated taking as study objects spatially-distinct microbiomes that share the historical contingency of local habitat genesis (i.e. geogenesis). This fundamental aspect links the ecological basis of this approach to the evolutionary continuous and dynamic trajectory of the soil formation.

A recent surge in the literature has highlighted the importance of understanding the relative contributions of microbial traits to community changes and how these are distributed across divergent community types^6,7^. Notably, functional traits can be measured at various levels of organization – from individual cells, to species, to whole communities^6^. The latter, denoted as ‘community-level traits’, can be quantified as community-aggregated traits (CATs), where traits are measured in a random sample of individuals irrespective of their taxonomic identities. Such a ‘top-down’ approach has proven to yield useful predictors of community-level properties, ranging from soil^8,9^ to marine waters^10^ to host-associated gut systems^11^.

**Figure 1:**
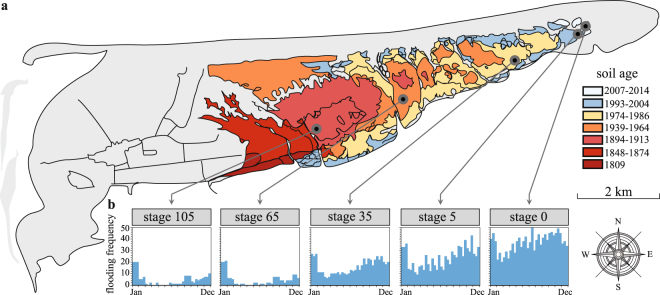
Map of the island of Schiermonnikoog, The Netherlands (N53°30′ E6°10′). (**a**) The chronology of the geogenesis along the gradual eastward extension of the island, dating from 1809 to 2014 reconstructed from topographic maps, aerial photos and ground-based vegetation maps. Different stages of soil formation were precisely located in the field (see Materials and Methods for details), named as stages 0, 5, 35, 65 and 105 (in years of soil development) (see refs^12–14^). (**b**) Bar charts depict the flooding frequency (i.e., number of times each plot was flooded) per month along the year of 2012 (scaled 0 to 50). The map was created using the software ArcGIS Pro v. 2.0 (http://pro.arcgis.com/en/pro-app/).

Here we interrogate whether and how progression in soil formation in an undisturbed marine-to-land gradient leads to shifts in the dominance of prevailing soil microbial community-traits. We thus investigated the microbiomes in a soil formation chronosequence at the island of Schiermonnikoog (The Netherlands), a pristine ecosystem that evolves with a trackable rate^12,13^. The chronosequence covers an area of approximately 8 km long that spans over a century of an ecosystem development^12,13^. The system has been carefully validated with respect to its space-for-time replacement, i.e. all stages of the chronosequence were found to have a similar temporal development^13,14^. In addition, stable isotope analyses (δ^13^C and δ^15^N) revealed a gradual shift from a system dominated by (external) marine carbon and nutrient inputs to a system dominated by (internal) terrestrial nutrient cycling^15^. In this system, geogenesis occurs through sequential sedimentation of silt and clay particles that are carried by the tides (Figs 1a and S1), resulting in a progressive eastward extension of the island^12^. Through time, the ongoing particle sedimentation causes a progressive decrease of flooding frequency and an increasingly finer soil texture, reducing the diffusion processes in the soil microhabitats (Fig. 1b). Flooding frequency, sodium level and resource availability (particularly dissolved organic carbon) were found to be key determinants of microbial community assembly along this chronosequence, leading to phylogenetically distinct microbiomes across sites^16,17^. Here, we hypothesize that the process of soil formation imposes variable selective pressures on the abundance of specific microbial CATs, leading to progressive shifts in the prevailing ecological strategies in the soil microbiomes. This hypothesis was built on the principle of dispersal-competition trade-off, which constitutes a fundamental ecological synthesis used to explain the coexistence of species in spatially-structured habitats^18,19^, ecological succession dynamics^20^, and abundance patterns of taxa^21^. Dispersal-competition trade-off may also direct the genetic diversity of microbial communities^22^, which often inhabit environments that are seemingly homogeneous but in fact highly structured at the micro-scale. Importantly, this ecological classification reflects similar ideas as the r versus K selection theory of MacArthur and Wilson^23^ and the ruderal versus the stress tolerance and competitive strategies of Grime^24^. Specifically, we explored the soil microbiomes with respect to community-level functional traits that match either the ‘dispersal’ (i.e. genes encoding chemotaxis and flagellar motility) or the ‘competition’ *modus* (i.e. genes encoding antibiotic resistance and complex substrate degradation) along an undisturbed gradient of soil formation.

## Results and Discussion

### Soil metagenomes: sequencing and annotation

To study a putative transition in dominant ecological strategies (that is, examining the ‘dispersal’ versus ‘competition’ *modus*), we used metagenomics data that were produced from the microbiomes sampled along the soil formation chronosequence. We thus extracted total DNA from triplicate soil samples (top 10 cm) along five distinct soil successional stages, i.e. the stages denoted as 0, 5, 35, 65 and 105 years of soil development (Supplementary Table S1). We then performed DNA-based high-throughput sequencing followed by computational sequence analyses. The sequenced metagenomes had sizes of 1.9 ± 0.7 (mean ± SD) gigabases (Gb). The raw sequences were quality-trimmed in MG-RAST^25^ and annotated using the KEGG Orthology (KO) identifiers^26^ (E ≤ 10^−5^) (ca. 12.6 ± 0.9% of the total reads), and custom-built profile hidden Markov model (HMM) databases for antibiotic resistance genes (ARGs) (E ≤ 10^−10^)^27^ (ca. 1 ± 0.1% of the total reads) and for carbohydrate-active enzymes (CAZy) (E ≤ 10^−5^)^28^ (ca. 0.8 ± 0.1% of the total reads). Details on the metagenome datasets are provided in Supplementary Table S2.

### Beta-diversity in microbiomes along the gradient of soil formation

**Figure 2:**
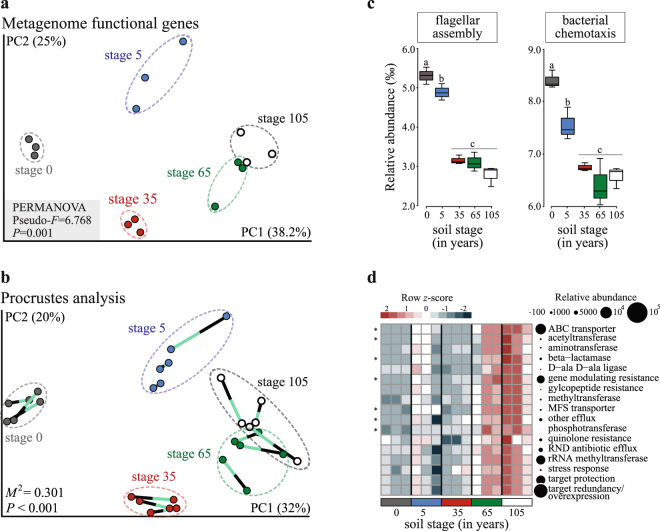
Functional metagenomes correlate with taxonomic composition across the stages of soil formation and the ‘dispersal’ and ‘competition’ response *modus*. (**a**) Principal coordinate analysis (PCoA) based on Bray-Curtis distances calculated from normalized KO annotations. (**b**) Procrustes analysis depicts a significant correlation between functional metagenomes (Bray-Curtis) and bacterial community composition (Bray-Curtis) across the soil stages. (**c**) Box plots display the abundance distribution of flagellar assembly (upper) and chemotaxis (down) mechanisms (based on KO annotations) of microbiomes across the soil stages (asterisks denote independent Mann-Whitney U test results performed only for pairwise comparisons between consecutive soil stages, *P < 0.05, **P < 0.001). (**d**) Heatmap displays the relative abundance (row *z*-scores) of ARGs by mechanism. ABC, ATP-binding cassette; MFS, major facilitator superfamily; RND, resistance-nodulation-cell division. Circles are proportional to the relative abundance of each ARG mechanism in all samples. Asterisks denote ARG mechanisms that differentially segregated across the soil stages, identified by random forest analysis with Boruta feature selection (average *z*-scores of 1000 runs >4) (Supplementary Table S5).

We calculated the differences between the taxonomic (using specific bacterial and fungal markers) and functional (KO, ARG, and CAZy) microbial community structures across the soil chronosequence. The data showed that the taxonomic and functional community structures changed systematically with increasing time of soil formation (Figs 2a and S2a,b, S4a and S5a; Supplementary Table S3), and that these changes were significantly correlated (Fig. 2b; *P* < 0.001). Both the functional (KO-based) profiles and the bacterial community compositions (16S rRNA gene-based) clustered according to soil developmental stage, as these consistently displayed highly significant goodness-of-fit measures (RELATE *ρ* = 0.782, *P* < 0.001; Fig. 2b). Similar results were obtained when the community compositions were compared with the soil resistomes (antibiotic resistance genes [ARG] counts — Supplementary Fig. S4b) and catabolic profiles (CAZy counts — Supplementary Fig. S5b,c and Supplementary Table S4) (Bray-Curtis distances). Collectively, these findings support an evolutionary coupling of phylogeny and function across the different soil microbiomes, and corroborate findings previously reported in the literature^8,27^.

### Evidence for dispersal-competition tradeoff in microbiomes during land colonization

In line with the ‘dispersal’ *modus*, we suggest that the diffusibility of compounds in the different microhabitats along the soil chronosequence was the key driver of the ability of organisms to either, withstand competition and/or predation and/or exploit the nutrients or migrate. The flooding frequency in the system decreased systematically along the course of succession (from ca. 37 ± 7 flooding events per month at the early stage, down to 5 ± 5 events at the later one; a full description, including seasonal tidal variations, is provided in Fig. 1b). Thus, we posit that, in the early-stage microhabitats, microorganisms depend on motility as a main life support trait, whereas we expect such traits to lose relevance in habitats in which compound diffusibility is constrained by structure, local viscosity or strain^29^. The *in silico* reconstructions of the genetic pathways for bacterial chemotaxis (PATH: ko02040) and flagellar assembly (PATH: ko02030), per community type across the soil formation gradient, revealed these pathways to be significantly overrepresented at the initial sites (stages 0 and 5), with their relative abundances steadily decreasing towards the late ones (Figs 2c and S3). Thus, in the less-structured soils from the initial stages, where daily tide movements generate constant water fluxes and turnover of high-quality resources (easily decomposable organic matter of marine origin), such traits are thought to assist the local populations in their exploration and exploitation of resource-rich or refuge-like microhabitats. For instance, while in non-turbulent conditions, non-motile cells can explore only ~80 nanoliters of water per day, motile cells can explore over 1 mililiter^30^. This has large implications for the speed at which nutrients are captured in pelagic habitats^30–32^. It is worth mentioning that, in spite of the fact that key groups of marine bacteria (e.g. SAR11 and *Prochlorococcus*) are non-motile, and as such depend exclusively on Brownian motion for movement, bacterial motility is clearly a widespread trait in microbes living in the ocean. Particularly in coastal seawater samples, the occurrence of this trait often ranges from <10 to up to 80% of the total community, varying depending on seasonality, type of system (rich in exploitable surfaces or not), and levels of particulate organic carbon^31,33^. Thus, the transient nutrient patches that occur in these habitats, representing resources that are available in limited space and time, exert a selective force on heterotrophs with respect to motility and chemotaxis traits. Another basis for the overrepresentation of such traits may lie in the quickly changing local conditions, for instance with respect to the levels of toxins, oxygen, redox potential, pH, osmolarity and the intensity and wavelength of light^34,35^. The overrepresentation of ‘dispersal-related’ pathways in the early stages of soil formation therefore likely reflects the intrinsic dynamics of these communities, which requires very quick responses to changing conditions. In these early successional habitats, the short-lived local patchy resources are highly dynamic, e.g. as a result of the filtration of pore water caused by the daily tides. In line with these arguments, our data also revealed a raised number of rRNA operons per organism at the early stages as compared to the later stages of land formation (see ref.^36^ for details). These data have been interpreted as a proxy for the ability of bacteria to deal with rapid changes, in particular, increases in nutrient levels. In addition, our findings are consistent with the argument that stochastic processes — leading to temporal niche partitioning — constitute a fundamental ecological mechanism explaining the assembly and turnover of the microbiomes at the early stages of soil formation^16,37^. The community assembly at these early successional sites is probably mainly governed by ecological dispersal. The importance of this process has also been highlighted in other primary and secondary successional gradients. For instance, both in a recently deglaciated soil chronosequence^38,39^ and in a post-mining field^40^, ecological dispersal was the main process contributing to the build-up (and restoration) of microbial community diversity at initial (or recently impacted) sites. At these early successional stages, the competition for resource uptake is often still low, and consequently, the effect of competitive exclusion is expected to be minor^41^.

**Figure 3:**
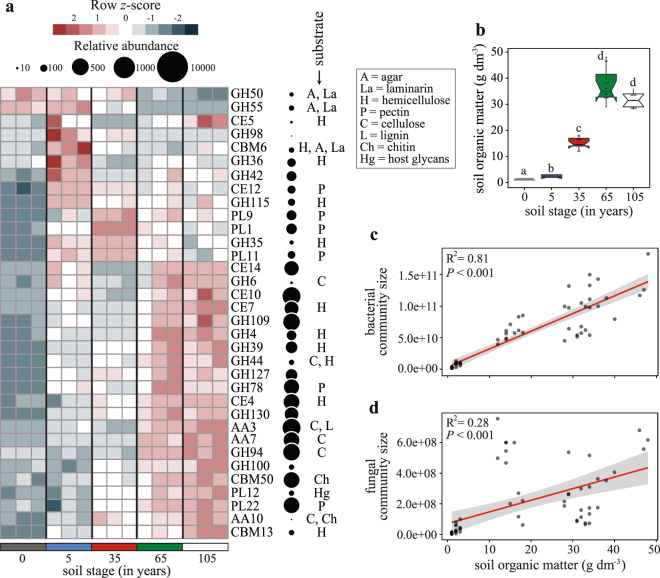
Soil organic matter (SOM) drives the size of the microbiomes and the distribution of CAZy families through soil formation. (**a**) Heatmap displays the relative abundance (row *z*-scores) of CAZy gene families that segregated statistically differently across the soil stages, identified by random forest analysis with Boruta feature selection (average *z*-scores of 1000 runs >4) (Supplementary Table S5). Circles are proportional to the relative abundance of each gene family in all samples. (**b**) Notched box plot depicts the increments in SOM across the soil stages. Different letters indicate significant differences (independent Mann-Whitney U test results performed only for pairwise comparisons between consecutive soil stages, *P* < 0.001). (**c**) Bacterial and (**d**) fungal community sizes (log copy numbers of the maker gene per gram of soil) increments through soil formation. Data are shown as a function of the progressive increments in SOM. The solid lines are linear regression models (contour lines are 95% confidence intervals), and statistics are provided on the panels.

The relative importance of the alternative ecological strategy — the ‘competition’ *modus* — was interrogated by profiling the ability of the microbiomes to resist antibiotics and to degrade complex organic compounds. We hypothesized these traits to be relevant in highly-structured environments, where microorganisms rely — to a greater extent — on chemical warfare for survival. Antibiotics can act as both antimicrobial and signaling molecules, either of these functions benefiting the producer under different conditions^29^. We here focused on the prevalence of resistance to naturally-produced antibiotics. Antibiotic resistance genes (ARGs) occur naturally in microbiomes and may have supported the primordial establishment of bacteria in soils^42^. We examined the prevalence of ARGs of different classes, including predicted drug transporters (e.g. major facilitator superfamily, ABC transporters), antimicrobial modification mechanisms (e.g. acetyltransferases, phosphotransferases) and inactivation mechanisms (e.g. beta-lactamase). The data indicated that, across the soil chronosequence, the ARG patterns had an opposite abundance distribution to that observed for the traits and pathways related to the ‘dispersal’ *modus* (Fig. 2d). This finding is consistent with our dispersal-competition tradeoff hypothesis, in which the increased soil patchiness towards the end of the succession leads to the selection of traits associated with chemical warfare, as opposed to the selection of traits associated with chemosensory motility that are more relevant in the early stages of soil formation. Moreover, the multiplicity of conditions in the soil microhabitats, with different resource levels, is thought to exert significant selective pressures on the local microorganisms with respect to how these deal with competition for local resources. This particular chronosequence is characterized by a progressive increment in soil organic matter (SOM) through time (ca. 2 ± 1 g dm^−3^ at early stages up to 35 ± 6 g dm^−3^ at later ones) (Fig. 3b), resulting from the sediment trapping and organic matter accumulation from the developing vegetation^12^. This increase in SOM was consistent with increments in both the overall bacterial and fungal community sizes (Fig. 3c,d), although more so for the first than for the second group (R^2^ = 0.81 and R^2^ = 0.28, *P* < 0.001, respectively). In line with the ‘competition’ *modus*, we thus expected a higher prevalence of genes associated with the degradation of complex compounds in the later stages of soil formation. Moreover, as SOM in the transition zones often consists of a mixture of marine and terrestrial organic inputs^15^, the metagenomes of the local microbiomes were expected to reflect such conditions. Indeed, different categories of carbohydrate metabolism traits were found to dominate along the chronosequence. For instance, traits (as reflected by genes of specific CAZy family proteins) involved in the degradation of labile C sources (e.g., GH50, GH55), often of marine origin in this system, such as agar and laminarin, prevailed in the early stages of the chronosequence. In contrast, traits involved in the degradation of complex plant-derived substrates, including pectin (e.g. PL1, PL9, PL11), and hemicellulose-degrading enzymes (GH35, GH115), dominated at the intermediate stage 35, and those CAZy oxidative enzymes at the late soil stages (e.g., AA3, AA7) (for details see Figs 3a and S5d). Thus, local carbohydrate composition was unquestionably a fundamental driver of community functionality and composition. Interestingly, the presence of traits that allow degradation of algal-associated compounds at the early stages was in line with the high abundance of genes associated with bacterial chemotaxis, as such motility systems are known to be stimulated by compounds like those released through algal exudation^33^.

### Methodological considerations

In the present study, we used a well-validated chronosequence to investigate the systematic changes in microbial CATs along a gradient of soil formation. On the basis of this present-day ecological analog, we provide evidence and theory with respect to how distinct ecological strategies change in importance through the course of soil colonization, thus highlighting shifts in local habitat diffusibility as an important adaptive force in the conquest of terrestrial surfaces by microbial life. Here, we realize that using a present-day chronosequence to explore historical eco-evolutionary transitions in microbiomes is a bold idea that requires a careful interpretation and further exploration. Furthermore, the use of chronosequences assumes that a series of soil sites originated from the same parental material differs solely on the ages since they were formed. This approach does not take into account how changes in environmental conditions and unpredictable disturbances may have interfered in the trajectory of ecological succession. As such, studies on soil chronosequences require a cautious validation of the space-for-time substitution assumption (see Materials and Methods for details). For an additional discussion on the use of chronosequences to investigate long-term ecological succession, see ref.^5^. Finally, the conclusions provided here are built upon relative abundances of selected genes obtained from metagenomics data. Thus, the patterns noted represent the relative investments of the collective microbiome members in the genetic potential for the ‘dispersal’ versus ‘competition’ response *modus*. By definition, ecological traits refer to phenotypic characteristics of organisms, and changes in relative abundances of genes found in metagenomes do not necessarily equate with changes in CATs. This implies that the use of this approach has inherent limitations to be considered across distinct study types and community functions. For instance, as previously mentioned^6^, genes encoding functions that exhibit a high degree of plasticity should be examined with care, as these may be more prone to be variable in space and time, and across distinct microbiome systems.

### Concluding remarks

We here provide, for the first time, compelling evidence for the contention that key CATs favoring either a ‘dispersal’ or a ‘competition’ prevailing lifestyle *modus* drive the adaptation of microbiomes along a marine-to-terrestrial transition system. It is important to notice that the fine ecological strategies behind these overall themes (i.e. the type of chemotaxis, antibiotic resistance and/or substrate use behavior) may be diverse at the level of individuals or populations. Future prospective studies may partition such mechanisms and evaluate their quantitative importance across divergent community types, thus providing refined insights into the microbial trait adaptive changes in marine-to-terrestrial transition zones^43^. Moreover, as the composition of the microbiomes differs between each successive soil stage in this system, the differences observed at the level of CATs may have been — to a large extent — caused by shifts in community composition, rather than adaptive changes driven by evolutionary processes. Finally, using a well-validated chronosequence of soil formation to investigate systematic changes in microbiomes is of unique value, as it takes into account the natural formation of the ecosystem, and, as such, accounts its historical contingency. This clearly reflects how biotic and abiotic constraining factors progressively change in the emerging soil gradient and how these changes are reflected in the signatures of CATs in the extant microbiomes. Such an understanding holds a novel, and still unexplored, perspective on how microbiomes are assembled in soils, highlighting a particular set of CATs that underpins their establishment and continuous trajectory in their quest for land colonization.

## Materials and Methods

### Study site

The black-barrier salt marsh at the island of Schiermonnikoog (N53°30′ E6°10′) represents one of the largest and well-preserved natural salt marshes in Western Europe. From the west to the east, this ecosystem develops along ~8 kilometers encompassing over a century of ecosystem progression^12^. This salt marsh chronosequence has been monitored and calibrated using topographic maps, aerial photographs and the thickness of the sediment layer accumulating on top of the underlying sand layer^12,13^. In addition, permanent plots have monitored the space-for-time replacement of this soil formation at different soil stages for more than 20 years^14^. For this study, five stages were accurately identified and estimated as 0, 5, 35, 65 and 105 years of soil development in 2012 (the sampling year) (referred in the main text and figures as ‘stages 0, 5, 35, 65, 105′). The initial soil sites (stages 0 and 5) are characterized by bare sand and sparsely vegetated soils, respectively. These stages are located in the western part of the chronosequence and are regularly flooded by seawater (Figs 1b and S1). The natural disturbance of the system has been suggested to act as an important mechanism promoting temporal turnover and stochastic assembly of microbial community establishment in these early soils stages^16,17,37^. In particular, the regular flooding acts as a continuous source of immigration of marine-derived microorganisms, in addition to the contribution exerted by the intermittent (initial) input of organic nutrient into the soil. As the ecosystem develops, the chronosequence progresses towards a terrestrial salt marsh by gradually decoupling from the marine environment^15^. Intermediate (stage 35) and late soil sites (stages 65 and 105) are located in elevated areas of the island due to the progressive accretion of mineral particles over time (i.e. silt and clay) — the so-called ‘geogenesis’, and, consequently, these sites are less subjected to the influence of tides. These are well-structured and vegetated soils, having their food-web driven mostly by internal nutrient cycling^15^.

### Soil sampling

Triplicate plots (5 × 5 m^2^) were established at each identified soil stage (separated 25 m from each other) at the same base of elevation — position at the initial elevation gradient on the bare sand flats with a base elevation of 1.16 m ± 2.2 cm (mean ± SE) above the Dutch ordinance level (NAP). Importantly, differences in the base of elevation reflect differences in inundation regimes, therefore having strong influences on the dynamics and the fate of the ecosystem progression^12^. Soil samples were collected in each plot by randomly taking 20 soil cores (5 cm diameter, 10 cm depth) using sterile techniques, to represent a composite sample. Samples were placed in a sterile plastic bag, sealed and transported to the laboratory (<24 h). All samples were sieved (4 mm mesh size) and stored at −20 °C for total DNA extraction and at 4 °C for physicochemical analysis. Soil samples were collected in May, July, September and November 2012. All samples (*n* = 60) were subject to microbial (bacterial and fungal) community size estimation (quantitative PCR), soil physicochemical characterization and community profiling (bacterial and fungal markers). Shotgun metagenomics was performed for samples collected in July (*n* = 15) 2012.

### Nucleic acid isolation

Total soil DNA was isolated from 0.5 g of initial material using the MoBio PowerSoil DNA isolation kit (MoBio Laboratories, Carlsbad, CA, USA). The manufacturer’s protocol was slightly modified by the addition of glass beads (diameter 0.1 mm; 0.25 g) to the soil slurries followed by three cycles of bead beating (mini-bead beater, BioSpec Products, Bartlesville, OK, USA) for 60 s. Obtained DNA samples were quantified using the Quant-iT PicoGreen dsDNA assay kit (Invitrogen, Carlsbad, CA, USA) on a TECAN infinite M200 Pro (Maennedorf, Switzerland) plate reader reading at 485 nm excitation and 530 nm emission. All samples were standardized at equal concentrations for further analysis.

### Soil physical and chemical characterization

Prior to soil physiochemical characterization, all soil samples were air-dried and sieved through a 100-mesh sieve. Physical (clay:silt:sand %) and chemical determinations of soil organic matter (SOM), nitrate (N-NO_3_^−^), ammonium (N-NH_4_^+^), sulphate (S-SO_4_^2−^), sodium (Na) and pH were performed individually per sample. These determinations were conducted in collaboration with the Laboratory of Soil Analysis at the “Luiz de Queiroz” College of Agriculture (Department of Soil Sciences, ESALQ/USP, Piracicaba, SP, Brazil) according to the methodology described by van Raij *et al*.^44^. Soil N-NH_4_^+^ and N-NO_3_^−^ concentrations were determined after extraction in 50 mL of 2 M KCl using 10 g of soil as initial material, according to the methodology described by Keeney and Nelson^45^.

### Flooding frequency estimation

The frequencies of flooding were calculated by comparing the seawater elevation (measured at the Schiermonnikoog harbor every 10 minutes during the year of 2012) to the natural elevation of each plot. In the Fig. 1b, the *y*-axis displays the number of times per month each plot (triplicate per site) was estimated to be naturally flooded by sea water (i.e. the number of times that seawater flooded and retracted in each plot — counted as numbers of independent flooding events).

### Shotgun metagenomics

Shotgun sequencing was conducted following the procedure described in the Illumina TrueSeq DNA sample preparation protocol. Aliquots of each DNA sample were mechanically sheared before entering the library generation. Libraries were size-selected to 170–180 bp using an agarose gel. Sequencing was performed at the Argonne National Laboratory in the Next Generation Sequencing Core (NGS) using a 2 × 100 bp sequencing run on the Illumina HiSeq2000. Raw, unassembled Illumina reads were paired, dereplicated and quality filtered in MG-RAST^25^. Putative open ready frames on the quality-controlled sequences were called using FragGeneScan^46^. Metagenomes were functionally annotated using BLASTX searches against the KEGG (Kyoto Encyclopedia of Genes and Genomes) Orthology (KO) identifiers^26^ with a maximum E-value cutoff of 10^−5^, a minimum percent identity cutoff of 60% and a minimum alignment length cutoff of 15. ARG annotation was performed by searching the amino acid sequence against a previously developed collection of custom-built resistance-gene-specific profile HMMs (http://dantaslab.wustl.edu/resfams)^27^, using hmmsearch in the HMMER3 software package^47^, with a maximum E*-*value of 10^−10^. To generate more encompassing counts of general resistance functions (for example, beta-lactamases), gene counts were summed across all annotations that clearly belonged to the parent function (for example, class A Beta-lactamases, metallo Beta-lactamases, TEM Beta-lactamases), informed by established ARG ontology^48^. CAZy annotation was performed by searching against the HMM profile-based database of carbohydrate-active enzymes obtained from dbCAN v.3^28^, with a maximum E-value of 10^−5^.

### Statistical analyses

Cross-soil comparisons were measured using Bray-Curtis similarities calculated based on normalized and square-root transformed count matrices of unique gene sequences per either KOs, ARGs or CAZy families. As the size of the metagenomic libraries varied stochastically by soil sample (Supplementary Table S2), raw counts were normalized to metagenomic library size to account for inconsistent sample depth, facilitating comparison between soils prior to Bray-Curtis calculations. PCoA and PERMANOVA^49^ were performed using the homonymous routines in PRIMER6+^50^. Significance levels calculated in PERMANOVA were determined with 10^3^ permutations. Correlations between resemblance matrices were determined using a non-parametric Mantel-type test implemented as the RELATE routine in PRIMER6+. Procrustes analyses (least-squares orthogonal mapping) were performed in QIIME^51^ using two related distance matrices as input. In brief, Procrustes analysis attempts to stretch and rotate the points in one matrix, such as points obtained by principal coordinates analysis (PCoA), to be as close as possible to points in the other matrix, thus preserving the relative distance between points within each matrix^52^. The significance of any Procrustes transformation was determined by comparing the measure of fit, *M*^2^, between matched-sample PCoA plots to a distribution of *M*^2^ values empirically determined from 10,000 label permutations. In each of the 10,000 permutations, the *M*^2^ value (the sum of squared distances between matched sample pairs) was recalculated and the original *M*^2^ value compared to the simulated distribution in order to compute a *P*-value. Because the *M*^2^ value is dependent on the sample size and data structure, it is generally not comparable across Procrustes transformations. Rather, *P*-values were used to compare different Procrustes plots.

Predicted KOs, ARG mechanisms and CAZy families that segregated significantly between soil stages were identified using random forest analysis^53^ with 1,000 trees followed by the Boruta algorithm for feature selection (average *z*-scores of 1000 runs >4)^54^. These analyses were carried out in R using packages randomForest v4.6–7 and Boruta v3.0, respectively. Heatmaps were constructed based on *z*-score transformed functional annotations to improve normality and homogeneity of variances. Linear regression modeling and Mann-Whitney U tests were performed using SPSS Statistics version 22 (SPSS, Armonk, NY, USA).

### Bacterial 16S rRNA gene analysis

Sequencing of the 16S rRNA gene was performed on a Roche 454 GS-FLX automated pyrosequencer running the titanium chemistry as previously described in detail^16^. Briefly, the bacterial 16S rRNA primer set 515f/1061r (targeting the V4–V6 regions) was used given their ability to generate accurate phylogenetic information^55^ and having few biases against any bacterial taxa^56^, generating approximately 550-bp reads. All downstream processing was performed in QIIME^51^, for details see ref.^16^.

Quantitative PCR was used to estimate differences in bacterial community size (i.e. as the number of bacterial 16S rRNA gene copies per gram of dry-weight soil) across the soil stages. Quantifications were carried out twice for each soil replicate in 25 µL reactions containing 12.5 µL Power SYBR Green PCR Master Mix (Applied Biosystems, Frankfurt, Germany), 0.5 µL of 20 mg mL^−1^ bovine serum albumin (Roche Diagnostics GmbH, Mannheim, Germany), 2 µL of each 10 mM primer (FP16S 5′-GGT AGT CYA YGC MST AAA CG-3′ and RP16S 5-GAC ARC CAT GCA SCA CCT G-3′) targeting the regions V5-V6 of the bacterial 16S rRNA gene^57^ and 10 ng of the sample DNA — amplifying a fragment of 263-bp. The thermal cycler protocol was 95 °C for 10 min, 40 cycles of 95 °C for 27 s, 62 °C for 1 min, 72 °C for 30 s, on the ABI Prism 7300 Cycler (Applied Biosystems). The specificity of the amplification products was confirmed by melting curve analysis, and the expected size of the amplified fragment was checked in a 1.5% agarose gel. A standard curve was generated over six orders of magnitude from 10^3^ to 10^8^ copies of template per assay (R^2^ = 0.99) using a plasmid containing the cloned 16S rRNA gene from *Serratia plymuthica*. The quantitative PCR efficiency (*E*) was calculated according to the equation $E=[{10}^{(-1/{\rm{slope}})}-1]$. Possible inhibitory effects of co-extracted humic compounds were checked by spiking samples with a range of known concentrations of the plasmid. No apparent inhibition was observed.

### Fungal ITS analysis

Sequencing of the ITS region of the fungal rRNA gene was performed on an Illumina MiSeq paired-end (2 × 250 bp) platform. We used the primer set ITS1f ^58^ and ITS2^59^, slightly modified for sequencing using the Illumina MiSeq (for a detailed description see ref.^60^). All downstream processing was performed using QIIME^51^ and USEARCH v7^61^. The full pipeline is available at, http://brmicrobiome.org (see ref.^17^).

Quantifications of fungal community sizes (i.e. as the number of fungal ITS copies per gram of dry-weight soil) were carried out twice for each soil sample in 25 µl reactions containing 12.5 µl Power SYBR Green PCR Master Mix (Applied Biosystems, Frankfurt, Germany), 1.25 µl of 20 mg mL^−1^ bovine serum albumin (Roche Diagnostics GmbH, Mannheim, Germany), 1.25 µl of each 10 µM primer (ITS1f 5′-TCC GTA GGT GAA CCT GCG G-3′ and 5.8S 5′-CGC TGC GTT CTT CAT CG-3′)^58,62^ and 10 ng of the sample DNA — amplifying a fragment of ~300-bp. The thermal cycler protocol was 95 °C for 10 min, 40 cycles of 95 °C for 1 min, 53 °C for 30 s, 72 °C for 1 min, on the ABI Prism 7300 Cycler (Applied Biosystems). The specificity of the amplification, the presence of inhibitory compounds and the qPCR efficiency were tested as described for bacteria (see above). The standard curve (R^2^ = 0.97) was generated using a plasmid containing the cloned ITS region from *Rhizoctonia solani*. No apparent inhibition was observed.

### Data availability

The metagenome data sets supporting the results in this article are publically available at the MG-RAST server with the accession numbers 4558897.3, 4558900.3, 4558903.3, 4558906.3, 4558909.3, 4558912.3, 4558915.3, 4558918.3, 4558921.3, 4558924.3, 4558927.3, 4558930.3, 4558933.3, 4558936.3, 4558939.3. The amplicon sequence data sets are available at the EBI ENA with accession numbers PRJEB22178 (ERP024520): bacterial 16S rRNA sequencing data; and PRJEB22169 (ERP024509): fungal ITS sequencing data.

## Electronic supplementary material

Supplementary Information
