# Supplementary material for: Dispersal-competition tradeoff in microbiomes in the quest for land colonization
Source: Sci Rep. 2018 Jun 21;8:9451. doi: 10.1038/s41598-018-27783-6 (PMC6013473; doi:10.1038/s41598-018-27783-6)
Supplement: Supplementary file 1 — Supplementary Information [file 41598_2018_27783_MOESM1_ESM.pdf]

Dispersal-competition tradeoff in microbiomes in the quest for land colonization

Authors: Francisco Dini-Andreote[1*], Jan Dirk van Elsas[1], Han Olff[2], Joana Falcão Salles[1]

Supplementary Information

Supplementary Tables

Supplementary Table S1. Location and physicochemical parameters measured for the samples collected along the salt marsh chronosequence at the island of Schiermonnikoog, The Netherlands. Sampling was performed in the referred months in the year of 2012.

	Soil stage (in years)																			
	**Stage 0**				**Stage 5**				**Stage 35**				**Stage 65**				**Stage 105**			
**Soil physical structure**																				
Silt (%)	2.7±0.5				2.9±0.1				22.3±0.4				44.5±2.2				49.0±0.7			
Clay (%)	5.0±0.0				5.0±0.0				23.4±1.5				36.2±1.3				37.7±0			
Sand (%)	92.3±0.5				92.1±0.1				54.3±1.5				19.3±1.0				13.3±0.7			
**Soil chemical parameters**	**Sampling time (in the year of 2012)**																			
	**May[a]**	**July[a,b]**	**Sept[a]**	**Nov[a]**	**May[a]**	**July[a,b]**	**Sept[a]**	**Nov[a]**	**May[a]**	**July[a,b]**	**Sept[a]**	**Nov[a]**	**May[a]**	**July[a,b]**	**Sept[a]**	**Nov[a]**	**May[a]**	**July[a,b]**	**Sept[a]**	**Nov[a]**
Soil water content (%)	11±1	11±1	12±1	13±1	7±1	7±1	10±1	13±0	36±2	38±2	36±3	38±1	48±1	46±2	45±3	56±4	38±2	35±1	35±1	44±3
pH	8.7±0.1	8.7±0.1	8.6±0.1	8.6±0.1	8.3±0.1	8.3±0.1	8.3±0.1	8.3±0.1	7.7±0.1	7.8±0.1	7.8±0.1	7.8±0.0	7.4±0.0	7.4±0.1	7.4±0.1	7.4±0.1	7.6±0.0	7.4±0.1	7.4±0.1	7.5±0.1
Soil organic matter (g dm^{-3})	1±0	1±0	1±0	1±0	2±1	2±1	2±0	3±1	17±1	15±1	14±1	15±1	47±1	38±2	33±1	33±2	32±2	31±1	34±2	28±1
Sulphate (mg dm^{-3})	16±3	13±1	95±4	75±4	32±4	43±5	180±4	203±9	670±13	130±12	113±10	53±4	704±5	550±26	452±23	67±11	539±13	423±12	498±5	416±26
Sodium (mg dm^{-3})	155±13	178±19	752±88	647±182	170±15	187±9	1248±38	1587±89	6836±240	3782±340	4799±457	3755±94	4533±384	6447±204	5888±346	5200±438	5188±624	3541±170	4654±731	5039±687
Total nitrogen (mg kg^{-1})	462±50	329±44	371±116	378±105	490±76	530±74	467±172	478±78	3232±150	3253±389	2606±539	2679±209	9604±1187	8132±1955	8580±1785	8276±1212	6300±1646	5833±3003	5693±2660	5087±2151
Ammonium (mg kg^{-1})	5±1	4±1	4±1	4±1	6±1	6±1	5±2	6±1	38±3	38±4	30±6	33±4	115±16	94±18	102±21	99±15	75±20	70±37	67±32	60±30
Nitrate (mg kg^{-1})	3±0	2±0	2±0	2±0	3±1	3±1	3±1	3±1	21±2	21±3	17±4	18±2	63±7	51±8	57±12	55±8	42±11	39±20	38±18	34±17

[a]Quantitative PCR (bacteria and fungi)
[a]Parallel amplicon bacterial 16S rRNA sequencing
[a]Parallel amplicon fungal ITS sequencing
[b]Shotgun metagenome sequencing

Supplementary Table S2. Characteristics of the metagenomes generated by Illumina HiSeq2000 for soil samples collected along the salt marsh chronosequence at the island of Schiermonnikoog, The Netherlands.

	Soil stage (in years)														
	**Stage 0**			**Stage 5**			**Stage 35**			**Stage 65**			**Stage 105**		
	**Plot A**	**Plot B**	**Plot C**	**Plot A**	**Plot B**	**Plot C**	**Plot A**	**Plot B**	**Plot C**	**Plot A**	**Plot B**	**Plot C**	**Plot A**	**Plot B**	**Plot C**
**Geographic coordinates**															
Latitude	53°30’18’’N	53°30’18’’N	53°30’18’’N	53°30’7’’N	53°30’7’’N	53°30’7’’N	53°29’59’’N	53°29’59’’N	53°29’59’’N	53°29’35’’N	53°29’35’’N	53°29’35’’N	53°28’56’’N	53°28’56’’N	53°28’56’’N
Longitude	6°19’51’’E	6°19’51’’E	6°19’51’’E	6°19’54’’E	6°19’54’’E	6°19’54’’E	6°18’57’’E	6°18’57’’E	6°18’57’’E	6°16’20’’E	6°16’20’’E	6°16’20’’E	6°14’3’’E	6°14’3’’E	6°14’3’’E
***Metagenomes**															
MG-RAST ID	4558897.3	4558900.3	4558903.3	4558906.3	4558909.3	4558912.3	4558915.3	4558918.3	4558921.3	4558924.3	4558927.3	4558930.3	4558933.3	4558936.3	4558939.3
Metagenome size (bp)	1 946 098 904	3 039 009 260	1 729 121 135	1 985 828 415	2 470 040 059	2 541 714 674	2 876 930 138	1 658 957 366	2 169 497 340	1 883 521 083	1 814 228 985	1 103 890 408	384 386 953	503 545 455	2 659 532 084
Average sequence length (bp)	156 ± 27	157 ± 28	159 ± 29	164 ± 32	159 ± 29	150 ± 24	156 ± 26	157 ± 28	155 ± 27	155 ± 27	165 ± 33	167 ± 32	170 ± 34	167 ± 34	156 ± 28
Number of sequences	12 450 878	19 321 585	10 839 811	12 107 696	15 534 513	16 934 258	18 408 001	10 533 784	13 947 451	12 123 756	10 969 050	6 599 703	2 258 688	2 999 891	16 987 073
GC content (%)	56 ± 12	56 ± 12	54 ± 12	56 ± 12	56 ± 12	56 ± 12	55 ± 11	54 ± 11	55 ± 11	57 ± 10	56 ± 11	55 ± 11	55 ± 11	57 ± 10	57 ± 10
Number of predicted ORFs	11 596 311	17 647 296	10 070 537	11 124 734	14 473 485	15 374 314	17 155 698	9 823 968	13 024 803	11 275 236	10 201 327	6 140 974	2 057 545	2 794 317	15 889 995
ORFs with predicted function	3 934 757	5 853 920	3 272 425	4 105 274	5 645 080	5 925 433	5 285 542	3 104 792	3 894 995	3 794 182	3 610 530	2 181 005	755 878	1 025 318	5 627 796
Bacteria (%)	96.1	95.8	95.7	97	97.5	97.5	95.5	95.6	95.8	96.7	97.2	96.9	97.1	97.1	97.1
Eukarya (%)	2.1	2.5	2.4	1.8	1.6	1.8	2.1	2.1	1.9	1.5	1.4	1.5	1.8	1.3	1.3
Archaea (%)	1.5	1.4	1.6	1.1	0.8	0.6	2.1	2	2.1	1.6	1.2	1.4	0.9	1.4	1.4
KO matches	1 565 010	2 391 486	1 293 459	1 618 989	2 173 227	2 387 195	2 065 112	1 202 620	1 526 363	1 486 222	1 392 578	835 141	298 105	396 573	2 188 639
CAZy matches	102 392	157 078	87 041	126 660	161 093	161 471	174 120	99 232	130 412	116 255	118 351	73 099	25 784	33 566	175 447
ARG matches	84 995	131 065	73 283	100 002	120 458	113 877	123 456	72 389	93 161	88 463	99 059	60 103	21 635	28 837	13 7611

[*]Post QC values

Supplementary Table S3. Overall and pairwise comparisons of KO, ARGs and CAZy community profiles across five soil stages analysed using PERMANOVA (Bray-Curtis distance) with 10^3 permutations. Abbreviations: MS, mean sum of squares; SS, sum of squares.

KEEG Orthology (KOs)					
**Source**	**d.f.**	**SS**	**MS**	**Pseudo-F**	***P*-value**
Soil stages	4	282.24	70.559	6.7683	0.001
Residuals	10	104.25	10.425		
Total	14	386.48			
**Average similarity between stages**					
	**Stage 0**	**Stage 5**	**Stage 35**	**Stage 65**	**Stage 105**
Stage 0	96.267				
Stage 5	92.361	95.262			
Stage 35	92.801	91.858	96.612		
Stage 65	91.003	92.525	93.597	95.363	
Stage 105	90.31	92.254	92.086	94.408	94.222
**ARGs (Soil resistome)**					
**Source**	**d.f.**	**SS**	**MS**	**Pseudo-F**	***P*-value**
Soil stages	4	330.33	82.583	7.1732	0.001
Residuals	10	115.13	11.513		
Total	14	445.46			
**Average similarity between stages**					
	**Stage 0**	**Stage 5**	**Stage 35**	**Stage 65**	**Stage 105**
Stage 0	97.537				
Stage 5	93.373	94.617			
Stage 35	95.919	93.428	97.635		
Stage 65	90.506	92.843	91.391	94.456	
Stage 105	88.677	91.458	89.337	94.691	94.097
**CAZy**					
**Source**	**d.f.**	**SS**	**MS**	**Pseudo-F**	***P*-value**
Soil stages	4	439.44	109.86	12.209	0.001
Residuals	10	89.979	8.9979		
Total	14	529.42			
**Average similarity between stages**					
	**Stage 0**	**Stage 5**	**Stage 35**	**Stage 65**	**Stage 105**
Stage 0	96.917				
Stage 5	89.661	95.625			
Stage 35	90.774	92.961	97.228		
Stage 65	87.841	92.526	93.286	95.157	
Stage 105	86.475	91.703	91.377	94.829	94.813
**Bacterial 16S rRNA**					
**Source**	**d.f.**	**SS**	**MS**	**Pseudo-F**	***P*-value**
Soil stages	4	20051	5012.7	3.9968	0.001
Residuals	10	12542	1254.2		
Total	14	32592			
**Average similarity between stages**					
	**Stage 0**	**Stage 5**	**Stage 35**	**Stage 65**	**Stage 105**
Stage 0	49.955				
Stage 5	30.826	46.589			
Stage 35	20.308	24.118	51.268		
Stage 65	21.179	27.235	37.319	48.475	
Stage 105	22.663	30.452	36.088	46.805	53.684
**Fungal ITS**					
**Source**	**d.f.**	**SS**	**MS**	**Pseudo-F**	***P*-value**
Soil stages	4	31007	7751.8	6.8323	0.001
Residuals	10	11346	1134.6		
Total	14	42353			
**Average similarity between stages**					
	**Stage 0**	**Stage 5**	**Stage 35**	**Stage 65**	**Stage 105**
Stage 0	43.268				
Stage 5	17.903	49.15			
Stage 35	15.766	18.295	57.567		
Stage 65	15.624	22.596	12.554	53.396	
Stage 105	15.112	22.053	11.167	34.472	60.832

Supplementary Table S4. Spearman's correlation (ρ values), determined via RELATE tests, relating pairwise community composition (bacterial 16S rRNA, fungal ITS) and metagenomic distances (KO, ARGs, CAZy) based on Bray-Curtis distances.

	ρ	*P*-value
**Bacterial 16S rRNA, KO**	0.782	0.001
**Bacterial 16S rRNA, ARG**	0.263	0.021
**Bacterial 16S rRNA, CAZy**	0.729	0.001
**Fungal ITS, CAZy**	0.556	0.001

Supplementary Table S5. Layer decision of KOs, ARG mechanisms and CAZy families identified using random forest analysis with 1,000 trees followed by the Boruta algorithm for feature selection (average *z*-scores of 1,000 runs > 4).

**KEEG Orthologs (KOs)**			
**KO level 3**	**KO ID**	**Layer decision**	**Layer importance**
Bacterial chemotaxis	K00575	Rejected	Inf
Bacterial chemotaxis	K03406	***Confirmed***	10.1505
Bacterial chemotaxis	K03407	***Confirmed***	12.9054
Bacterial chemotaxis	K03408	***Confirmed***	8.8730
Bacterial chemotaxis	K03412	***Confirmed***	8.7897
Bacterial chemotaxis	K03413	***Confirmed***	10.5524
Bacterial chemotaxis	K05874	***Confirmed***	11.9026
Bacterial chemotaxis	K05875	***Confirmed***	8.3797

**KEEG Orthologs (KOs)**			
**KO level 3**	**KO ID**	**Layer decision**	**Layer importance**
Flagellar assembly	K02387	Rejected	Inf
Flagellar assembly	K02388	Tentative	3.7512
Flagellar assembly	K02389	Rejected	Inf
Flagellar assembly	K02390	***Confirmed***	4.8603
Flagellar assembly	K02391	***Confirmed***	6.2569
Flagellar assembly	K02392	***Confirmed***	4.2732
Flagellar assembly	K02393	Rejected	Inf
Flagellar assembly	K02394	***Confirmed***	5.9072
Flagellar assembly	K02396	***Confirmed***	4.5166
Flagellar assembly	K02397	Rejected	Inf
Flagellar assembly	K02400	Rejected	Inf
Flagellar assembly	K02401	Tentative	3.9386
Flagellar assembly	K02406	***Confirmed***	6.7124
Flagellar assembly	K02407	***Confirmed***	5.6035
Flagellar assembly	K02408	***Confirmed***	7.8791
Flagellar assembly	K02409	***Confirmed***	4.5325
Flagellar assembly	K02410	***Confirmed***	5.6311
Flagellar assembly	K02411	***Confirmed***	4.7666
Flagellar assembly	K02412	***Confirmed***	5.6361
Flagellar assembly	K02413	Rejected	Inf
Flagellar assembly	K02414	Rejected	Inf
Flagellar assembly	K02416	Tentative	4.0559
Flagellar assembly	K02417	***Confirmed***	5.8813
Flagellar assembly	K02418	Rejected	Inf
Flagellar assembly	K02419	Rejected	Inf
Flagellar assembly	K02420	Rejected	Inf
Flagellar assembly	K02421	Tentative	3.8170
Flagellar assembly	K02556	Rejected	Inf
Flagellar assembly	K02557	***Confirmed***	5.4375

**ARGs (Soil resistomes)**		
**Mechanism**	**Layer decision**	**Layer importance**
ABC transporter	***Confirmed***	7.1482
Acetyltransferase	***Confirmed***	6.9241
Aminotransferase	Rejected	Inf
Beta-lactamase	***Confirmed***	6.8547
D-ala D-ala ligase	Rejected	Inf
Gene modulating resistance	***Confirmed***	5.6460
Gylcopeptide resistance	Rejected	Inf
Methyltransferase	Rejected	Inf
MFS transporter	***Confirmed***	8.7414
Other efflux	***Confirmed***	4.9721
Phosphotransferase	***Confirmed***	7.8383
Quinolone resistance	Rejected	Inf
RND antibiotic efflux	Rejected	Inf
rRNA methyltransferase	Rejected	Inf
Stress response	Rejected	Inf
Target protection	Rejected	Inf
Target redundancy/overexpression	Rejected	Inf

CAZymes			
**Class**	**Gene family**	**Layer decision**	**Layer importance**
AA	AA1	Tentative	2.8021
AA	AA10	***Confirmed***	3.5571
AA	AA2	Rejected	Inf
AA	AA3	***Confirmed***	3.7742
AA	AA4	Tentative	2.9352
AA	AA5	Rejected	Inf
AA	AA6	Rejected	Inf
AA	AA7	***Confirmed***	3.4470
AA	AA8	Rejected	Inf
AA	AA9	Rejected	Inf
CBM	CBM1	Rejected	Inf
CBM	CBM10	Rejected	Inf
CBM	CBM11	Rejected	Inf
CBM	CBM12	Rejected	Inf
CBM	CBM13	***Confirmed***	4.1255
CBM	CBM14	Rejected	Inf
CBM	CBM15	Rejected	Inf
CBM	CBM16	Rejected	Inf
CBM	CBM17	Rejected	Inf
CBM	CBM18	Rejected	Inf
CBM	CBM19	Rejected	Inf
CBM	CBM2	Rejected	Inf
CBM	CBM20	Rejected	Inf
CBM	CBM21	Rejected	Inf
CBM	CBM22	Rejected	Inf
CBM	CBM23	Rejected	Inf
CBM	CBM25	Rejected	Inf
CBM	CBM26	Rejected	Inf
CBM	CBM27	Rejected	Inf
CBM	CBM28	Rejected	Inf
CBM	CBM29	Rejected	Inf
CBM	CBM3	Rejected	Inf
CBM	CBM30	Rejected	Inf
CBM	CBM31	Rejected	Inf
CBM	CBM32	Rejected	Inf
CBM	CBM34	Rejected	Inf
CBM	CBM35	Rejected	Inf
CBM	CBM36	Rejected	Inf
CBM	CBM37	Rejected	Inf
CBM	CBM38	Rejected	Inf
CBM	CBM39	Rejected	Inf
CBM	CBM4	Rejected	Inf
CBM	CBM40	Rejected	Inf
CBM	CBM41	Rejected	Inf
CBM	CBM42	Rejected	Inf
CBM	CBM43	Rejected	Inf
CBM	CBM44	Rejected	Inf
CBM	CBM45	Rejected	Inf
CBM	CBM46	Rejected	Inf
CBM	CBM47	Rejected	Inf
CBM	CBM48	Rejected	Inf
CBM	CBM49	Rejected	Inf
CBM	CBM5	Rejected	Inf
CBM	CBM50	***Confirmed***	3.5038
CBM	CBM51	Rejected	Inf
CBM	CBM52	Rejected	Inf
CBM	CBM53	Rejected	Inf
CBM	CBM54	Rejected	Inf
CBM	CBM55	Rejected	Inf
CBM	CBM56	Rejected	Inf
CBM	CBM57	Rejected	Inf
CBM	CBM58	Rejected	Inf
CBM	CBM59	Rejected	Inf
CBM	CBM6	***Confirmed***	3.9650
CBM	CBM60	Rejected	Inf
CBM	CBM61	Rejected	Inf
CBM	CBM62	Tentative	3.0064
CBM	CBM63	Rejected	Inf
CBM	CBM64	Rejected	Inf
CBM	CBM65	Rejected	Inf
CBM	CBM66	Rejected	Inf
CBM	CBM67	Tentative	2.9440
CBM	CBM8	Rejected	Inf
CBM	CBM9	Rejected	Inf
CE	CE1	Rejected	Inf
CE	CE10	***Confirmed***	3.2091
CE	CE11	Rejected	Inf
CE	CE12	***Confirmed***	4.1835
CE	CE13	Rejected	Inf
CE	CE14	***Confirmed***	3.5636
CE	CE15	Tentative	2.9137
CE	CE16	Rejected	Inf
CE	CE2	Tentative	3.0742
CE	CE3	Rejected	Inf
CE	CE4	***Confirmed***	3.3585
CE	CE5	***Confirmed***	3.8010
CE	CE6	Rejected	Inf
CE	CE7	***Confirmed***	3.2828
CE	CE8	Rejected	Inf
CE	CE9	Rejected	Inf
GH	GH1	Rejected	Inf
GH	GH10	Tentative	3.0285
GH	GH100	***Confirmed***	3.8906
GH	GH101	Rejected	Inf
GH	GH102	Rejected	Inf

GH	GH103	Rejected	Inf
GH	GH104	Rejected	Inf
GH	GH105	Rejected	Inf
GH	GH106	Rejected	Inf
GH	GH107	Rejected	Inf
GH	GH108	Rejected	Inf
GH	GH109	***Confirmed***	3,3174
GH	GH11	Rejected	Inf
GH	GH110	Rejected	Inf
GH	GH111	Rejected	Inf
GH	GH112	Rejected	Inf
GH	GH113	Rejected	Inf
GH	GH114	Rejected	Inf
GH	GH115	***Confirmed***	3,1135
GH	GH116	Rejected	Inf
GH	GH117	Rejected	Inf
GH	GH118	Rejected	Inf
GH	GH119	Rejected	Inf
GH	GH12	Rejected	Inf
GH	GH120	Rejected	Inf
GH	GH121	Rejected	Inf
GH	GH122	Rejected	Inf
GH	GH123	Rejected	Inf
GH	GH124	Rejected	Inf
GH	GH125	Rejected	Inf
GH	GH126	Rejected	Inf
GH	GH127	***Confirmed***	3.4860
GH	GH128	Rejected	Inf
GH	GH129	Rejected	Inf
GH	GH13	Rejected	Inf
GH	GH130	***Confirmed***	3.7615
GH	GH131	Rejected	Inf
GH	GH132	Rejected	Inf
GH	GH14	Rejected	Inf
GH	GH15	Rejected	Inf
GH	GH16	Rejected	Inf
GH	GH17	Rejected	Inf
GH	GH18	Rejected	Inf
GH	GH19	Rejected	Inf
GH	GH2	Tentative	2.8451
GH	GH20	Rejected	Inf
GH	GH22	Rejected	Inf
GH	GH23	Rejected	Inf
GH	GH24	Rejected	Inf
GH	GH25	Rejected	Inf
GH	GH26	Rejected	Inf
GH	GH27	Rejected	Inf
GH	GH28	Rejected	Inf
GH	GH29	Tentative	3.0446
GH	GH3	Rejected	Inf
GH	GH30	Tentative	3.0678
GH	GH31	Rejected	Inf
GH	GH32	Rejected	Inf
GH	GH33	Rejected	Inf
GH	GH35	***Confirmed***	4.0238
GH	GH36	***Confirmed***	3.8866
GH	GH37	Rejected	Inf
GH	GH38	Rejected	Inf
GH	GH39	***Confirmed***	3.8047
GH	GH4	***Confirmed***	3.3554
GH	GH42	***Confirmed***	3.4475
GH	GH43	Rejected	Inf
GH	GH44	***Confirmed***	3.1113
GH	GH45	Rejected	Inf
GH	GH46	Rejected	Inf
GH	GH47	Rejected	Inf
GH	GH48	Rejected	Inf
GH	GH49	Rejected	Inf
GH	GH5	Rejected	Inf
GH	GH50	***Confirmed***	3.4836
GH	GH51	Rejected	Inf
GH	GH52	Rejected	Inf
GH	GH53	Rejected	Inf
GH	GH54	Tentative	3.0008
GH	GH55	***Confirmed***	3.5308
GH	GH56	Rejected	Inf
GH	GH57	Rejected	Inf
GH	GH58	Rejected	Inf
GH	GH59	Rejected	Inf
GH	GH6	***Confirmed***	3.2089
GH	GH62	Rejected	Inf
GH	GH63	Rejected	Inf
GH	GH64	Rejected	Inf
GH	GH65	Rejected	Inf
GH	GH66	Rejected	Inf
GH	GH67	Rejected	Inf
GH	GH68	Rejected	Inf
GH	GH7	Rejected	Inf
GH	GH70	Rejected	Inf
GH	GH71	Rejected	Inf
GH	GH72	Rejected	Inf
GH	GH73	Rejected	Inf
GH	GH74	Rejected	Inf
GH	GH75	Rejected	Inf
GH	GH76	Rejected	Inf
GH	GH77	Rejected	Inf
GH	GH78	***Confirmed***	3.7183
GH	GH79	Rejected	Inf
GH	GH8	Rejected	Inf

GH	GH80	Rejected	Inf
GH	GH81	Rejected	Inf
GH	GH82	Rejected	Inf
GH	GH83	Rejected	Inf
GH	GH84	Rejected	Inf
GH	GH85	Rejected	Inf
GH	GH86	Rejected	Inf
GH	GH87	Rejected	Inf
GH	GH88	Rejected	Inf
GH	GH89	Tentative	2.8080
GH	GH9	Rejected	Inf
GH	GH90	Rejected	Inf
GH	GH91	Rejected	Inf
GH	GH92	Rejected	Inf
GH	GH93	Rejected	Inf
GH	GH94	***Confirmed***	3.3032
GH	GH95	Rejected	Inf
GH	GH96	Rejected	Inf
GH	GH97	Rejected	Inf
GH	GH98	***Confirmed***	3.1358
GH	GH99	Rejected	Inf
GT	GT1	***Confirmed***	3.1364
GT	GT10	***Confirmed***	3.5962
GT	GT11	Rejected	Inf
GT	GT12	Rejected	Inf
GT	GT13	Rejected	Inf
GT	GT14	Rejected	Inf
GT	GT15	Rejected	Inf
GT	GT16	Rejected	Inf
GT	GT17	Rejected	Inf
GT	GT18	Rejected	Inf
GT	GT19	Rejected	Inf
GT	GT2	Rejected	Inf
GT	GT20	***Confirmed***	3.0824
GT	GT21	Rejected	Inf
GT	GT22	Rejected	Inf
GT	GT23	Rejected	Inf
GT	GT24	Tentative	2.7951
GT	GT25	***Confirmed***	4.2333
GT	GT26	Rejected	Inf
GT	GT27	Rejected	Inf
GT	GT28	Rejected	Inf
GT	GT29	Rejected	Inf
GT	GT3	Rejected	Inf
GT	GT30	Rejected	Inf
GT	GT31	Rejected	Inf
GT	GT32	Rejected	Inf
GT	GT33	Rejected	Inf
GT	GT34	Rejected	Inf
GT	GT35	***Confirmed***	3.3479
GT	GT37	Rejected	Inf
GT	GT38	Rejected	Inf
GT	GT39	Rejected	Inf
GT	GT4	***Confirmed***	3.1818
GT	GT40	Rejected	Inf
GT	GT41	Rejected	Inf
GT	GT42	Rejected	Inf
GT	GT43	Rejected	Inf
GT	GT44	Rejected	Inf
GT	GT45	Rejected	Inf
GT	GT46	***Confirmed***	3.6020
GT	GT47	***Confirmed***	3.1583
GT	GT48	Rejected	Inf
GT	GT49	Rejected	Inf
GT	GT5	Rejected	Inf
GT	GT50	Rejected	Inf
GT	GT51	***Confirmed***	3.3192
GT	GT52	Rejected	Inf
GT	GT53	Confirmed	3.2380
GT	GT54	Rejected	Inf
GT	GT55	***Confirmed***	3.3647
GT	GT56	Rejected	Inf
GT	GT57	Rejected	Inf
GT	GT58	Rejected	Inf
GT	GT59	Rejected	Inf
GT	GT6	Rejected	Inf
GT	GT60	***Confirmed***	3.3647
GT	GT61	Rejected	Inf
GT	GT62	Rejected	Inf
GT	GT63	Rejected	Inf
GT	GT64	Rejected	Inf
GT	GT65	Rejected	Inf
GT	GT66	Tentative	3.0183
GT	GT67	Rejected	Inf
GT	GT68	Rejected	Inf
GT	GT69	Rejected	Inf
GT	GT7	Rejected	Inf
GT	GT70	Rejected	Inf
GT	GT71	Rejected	Inf
GT	GT72	Rejected	Inf
GT	GT73	Rejected	Inf
GT	GT74	Tentative	3.0000
GT	GT75	Rejected	Inf
GT	GT76	Rejected	Inf
GT	GT77	Rejected	Inf
GT	GT78	Rejected	Inf
GT	GT79	Rejected	Inf
GT	GT8	Rejected	Inf
GT	GT80	Rejected	Inf

GT	GT81	Rejected	Inf
GT	GT82	Rejected	Inf
GT	GT83	Tentative	2.9641
GT	GT84	Rejected	Inf
GT	GT85	Rejected	Inf
GT	GT87	Rejected	Inf
GT	GT88	Rejected	Inf
GT	GT89	Rejected	Inf
GT	GT9	Rejected	Inf
GT	GT90	Rejected	Inf
GT	GT91	Rejected	Inf
GT	GT92	Rejected	Inf
GT	GT93	Tentative	2.8188
GT	GT94	Rejected	Inf
PL	PL1	***Confirmed***	3.8243
PL	PL10	Rejected	Inf
PL	PL11	***Confirmed***	4.6552
PL	PL12	***Confirmed***	3.6877
PL	PL13	Rejected	Inf
PL	PL14	Rejected	Inf
PL	PL15	Rejected	Inf
PL	PL16	Rejected	Inf
PL	PL17	Rejected	Inf
PL	PL18	Rejected	Inf
PL	PL2	Rejected	Inf
PL	PL20	Rejected	Inf
PL	PL21	Rejected	Inf
PL	PL22	***Confirmed***	3.2002
PL	PL3	Rejected	Inf
PL	PL4	Rejected	Inf
PL	PL5	Rejected	Inf
PL	PL6	Rejected	Inf
PL	PL7	Rejected	Inf
PL	PL8	Rejected	Inf
PL	PL9	***Confirmed***	3.3642

Supplementary Figures

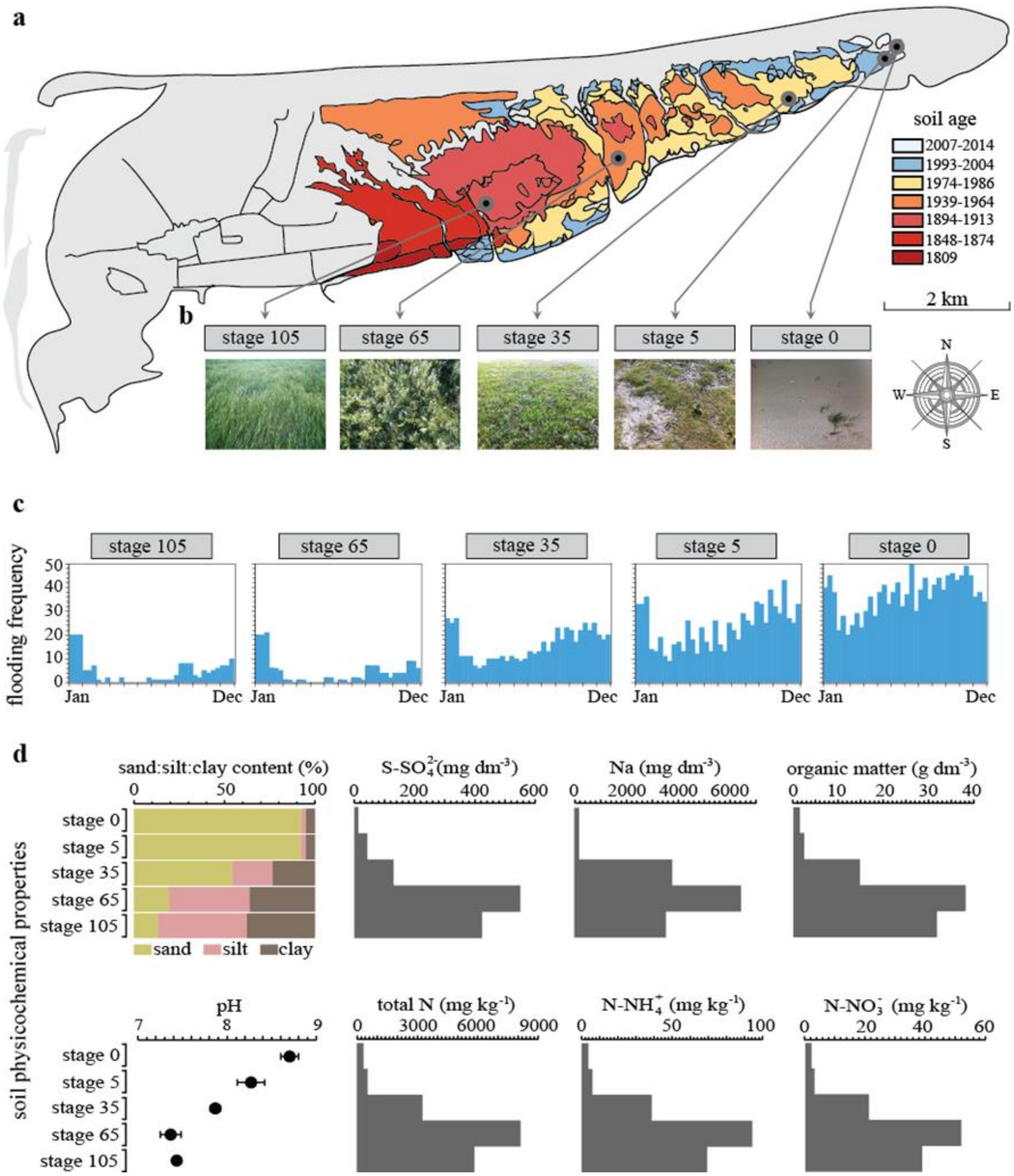

Supplementary Fig. S1. Map of the island of Schiermonnikoog, the Netherlands (N53°30' E6°10') and characteristics of the soil stages across the chronosequence. (a) The scaled map depicts the chronology of the geogenesis along the gradual eastward extension of the island, dating from 1809 to 2014. Throughout the development of this ecosystem, different stages of soil formation were precisely located (see Materials and Methods), named as stages 0, 5, 35, 65 and 105 (in years of soil development). **(b)** Photographs of each plot taken in July 2012. **(c)** Bar charts depict the flooding frequency (i.e., number of times each plot was flooded) per month along the year of 2012 (scaled 0 to 50). **(d)** Physicochemical data display soil sand:silt:clay content (%), sulphate (S-SO_4^{2-}), sodium (Na), soil organic matter (SOM), total nitrogen (N), ammonium (N-NH_4^+), nitrate (N-NO_3^-), and pH — at the five stages of soil formation. The map was created using the software ArcGIS Pro v. 2.0 (http://pro.arcgis.com/en/pro-app/).

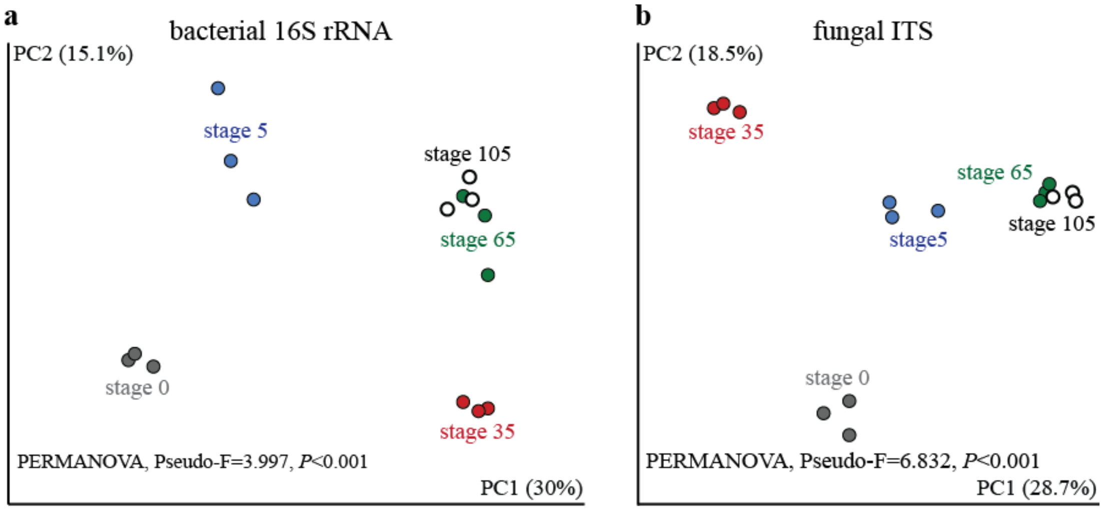

Supplementary Fig. S2. Differences in bacterial and fungal β-diversities across the stages of soil formation. (a) Bacterial and **(b)** fungal principal coordinate analyses (PCoA) based on Bray-Curtis distances calculated using the bacterial 16S rRNA and the fungal ITS amplicon sequencing data, respectively. PERMANOVA was performed using the homonymous routines in PRIMER6+ with 10^3 permutations (Supplementary Table S3).

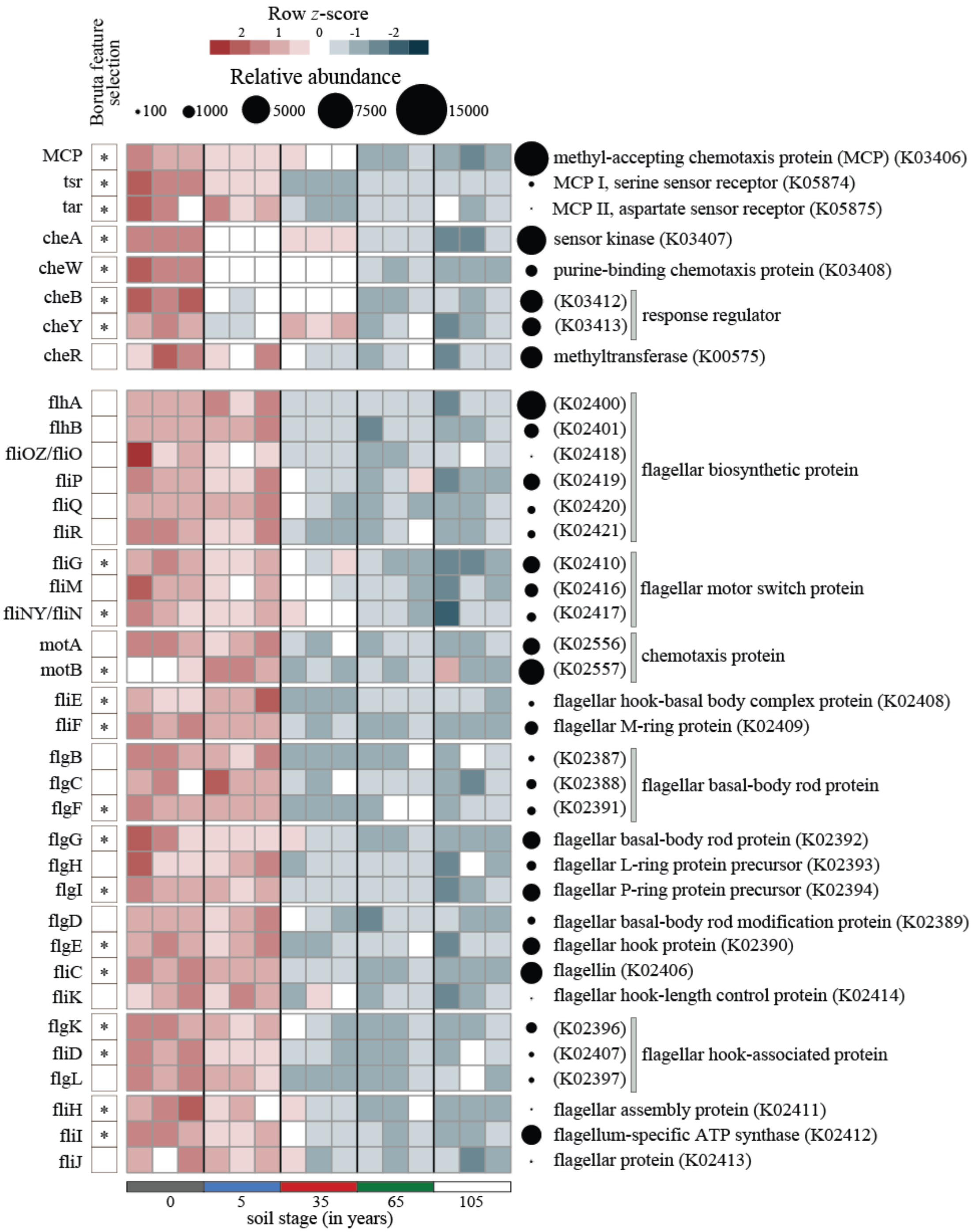

Supplementary Fig. S3. Distribution of KOs involved in bacterial chemotaxis and flagellar assembly across the stages of soil formation – the 'dispersal' *modus*. Heatmap displays the relative abundance (row *z*-scores) of the KOs involved in bacterial chemotaxis and flagellar assembly. Circles are proportional to the relative abundance of each KO in all samples. Asterisks denote KOs that differentially segregated across the soil stages, identified by random forest analysis with Boruta feature selection (average *z*-scores of 1000 runs > 4) (Supplementary Table S5).

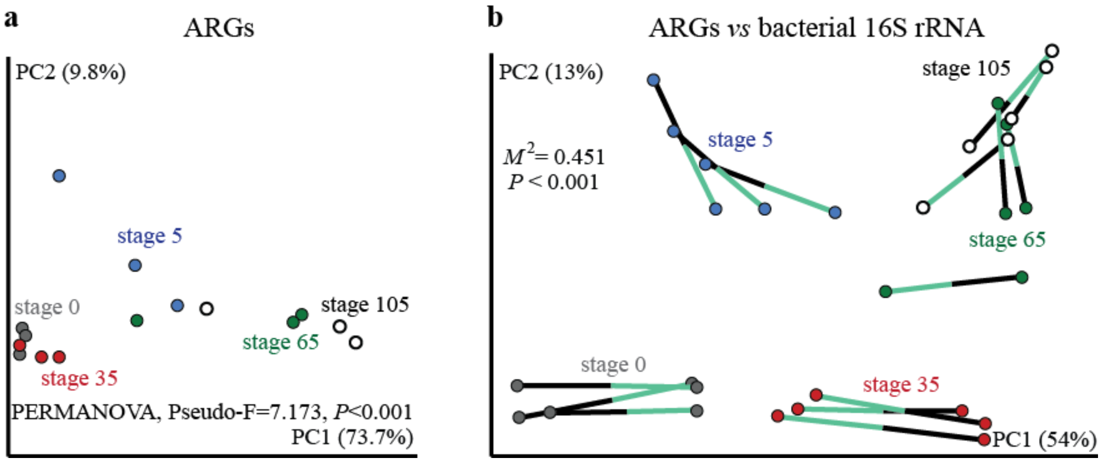

Supplementary Fig. S4. Patterns of soil resistomes across the stages of soil formation. (a) Principal coordinate analysis (PCoA) based on Bray-Curtis distances calculated from normalized ARG annotations. PERMANOVA was performed using the homonymous routines in PRIMER6+ with 10^3 permutations (Supplementary Table S3). **(b)** Procrustes analysis depicts the significant correlation between soil resistomes (Bray-Curtis) and bacterial community composition (Bray-Curtis) across the soil stages.

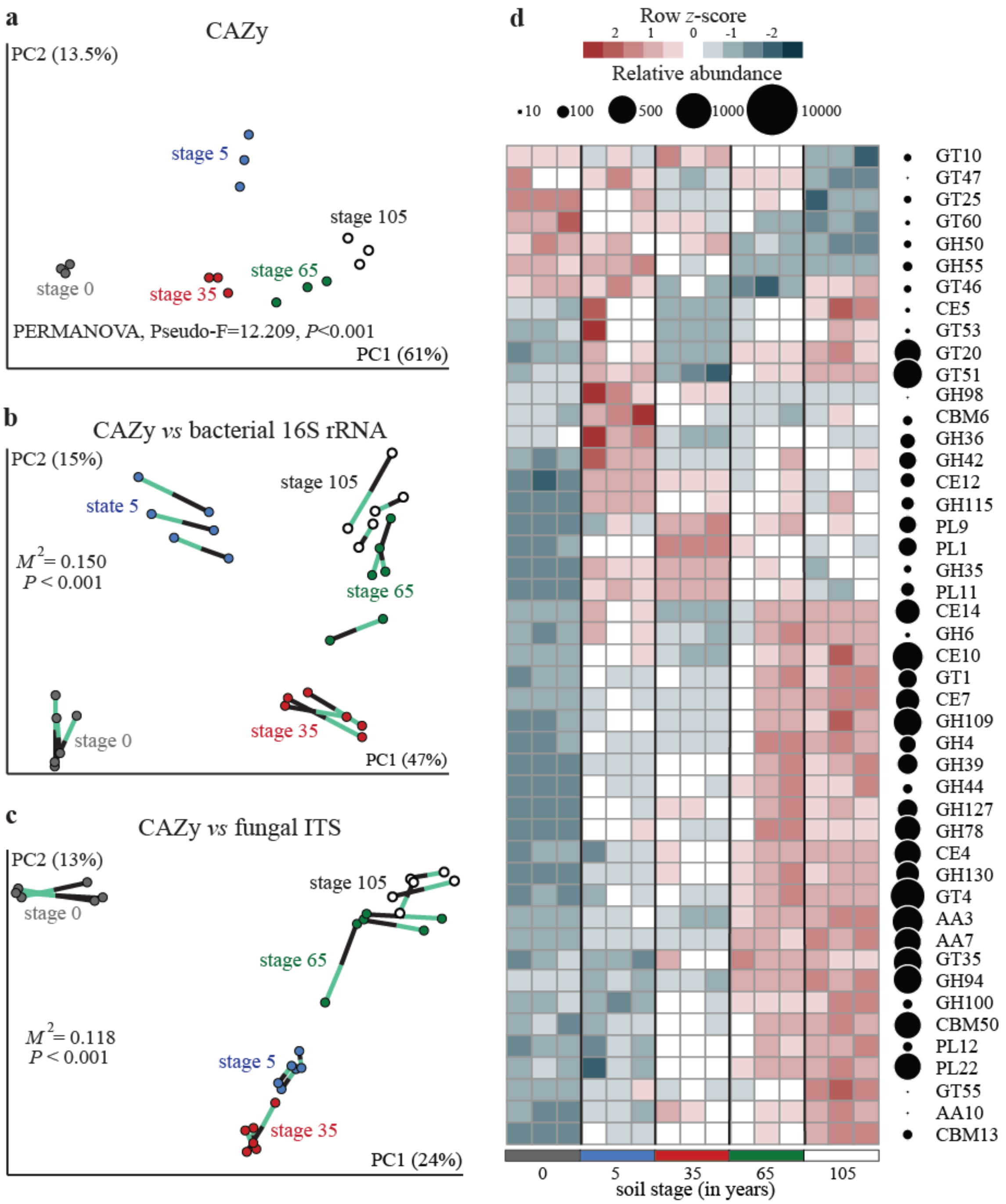

Supplementary Fig. S5. CAZy profiles across the stages of soil formation. (a) Principal coordinate analysis (PCoA) based on Bray-Curtis distances calculated from normalized CAZy family annotations. PERMANOVA was performed using the homonymous routines in PRIMER6+ with 10^3 permutations (Supplementary Table S3). Procrustes analyses depict significant correlations between CAZy profiles (Bray-Curtis) and bacterial **(b)** and fungal **(c)** community compositions (Bray-Curtis) across the soil stages. **(d)** Extended version of Fig. 3a that includes glycosyltransferase (GT) families. Heatmap displays the relative abundance (row *z*-scores) of CAZy gene families that differentially segregated across the soil stages, identified by random forest analysis with Boruta feature selection (average *z*-scores of 1000 runs > 4) (Supplementary Table S5). Circles are proportional to the relative abundance of each gene family in all samples.
